# Successful Treatment of Community-Acquired Methicillin-Resistant Staphylococcus Aureus Necrotizing Pneumonia in the Setting of Chronic Graft-Versus-Host Disease

**DOI:** 10.7759/cureus.13123

**Published:** 2021-02-04

**Authors:** Kavita Renduchintala, Sowmya Nanjappa, Asha Ramsakal, John Greene

**Affiliations:** 1 Internal Medicine, Moffitt Cancer Center, Tampa, USA; 2 Infectious Diseases, University of Pittsburgh Medical Center, Pittsburgh, USA; 3 Infectious Diseases, Moffitt Cancer Center, Tampa, USA

**Keywords:** necrotizing pneumonia, methicillin resistant staphylococcus aureus (mrsa), graft versus host response

## Abstract

Necrotizing pneumonia (NP) is a rare complication of community-acquired pneumonia that results in tissue necrosis and permanent destruction of the lung parenchyma.

This study presents a case of a 21-year old male patient with T-cell acute lymphoblastic lymphoma who was treated with chemotherapy and matched-unrelated donor stem cell transplantation. His post-transplant course included chronic graft-versus-host disease (GVHD) and subsequent community-acquired methicillin-resistant Staphylococcus aureus (CA-MRSA) necrotizing pneumonia. In addition to antibiotics, steroids were used to help blunt the proinflammatory response following CA-MRSA pneumonia and this led to a rapid improvement in our patient’s clinical course.

CA-MRSA pneumonia is often treated with vancomycin. Given the nature of necrotizing pneumonia, the use of a toxin reducing agent like linezolid and adjunct therapy with corticosteroids was beneficial in the management of this disease process in our patient with chronic GVHD. Further prospective studies are needed to evaluate this regimen as a therapeutic alternative.

## Introduction

Necrotizing pneumonia is a rare complication of community-acquired pneumonia, in which toxin-producing bacteria activate an intense local inflammatory response that results in tissue necrosis and permanent destruction of the lung parenchyma, leaving cavities or pneumatoceles. Necrotizing pneumonia is usually caused by Staphylococcus aureus, Pseudomonas aeruginosa, Klebsiella pneumoniae, and anaerobic bacteria, and its symptoms include productive cough, hemoptysis, fever, and chills. Furthermore, necrotizing pneumonia is associated with pulmonary inflammation, cavitation, peripheral necrosis, and rapidly deteriorating respiratory function. 

Initial treatment for necrotizing pneumonia is broad-spectrum antibiotics, administered until an organism is isolated; antibiotic treatment is then tailored accordingly. Steroids are not routinely recommended. While some randomized controlled trials have shown that steroids can reduce mortality and decrease the length of stay, others have not shown significant differences in clinically important endpoints [1]. Therefore, guidelines at this time do not routinely recommend steroids to treat community-acquired pneumonia, and there are no specific treatment guidelines about necrotizing pneumonia in the setting of chronic graft-versus-host disease (GVHD).

## Case presentation

This study presents a case of a 21-year-old male patient with T-cell acute lymphoblastic lymphoma status post induction chemotherapy with lineage switch to acute myelogenous leukemia. He received high-dose chemotherapy with cyclophosphamide, total body irradiation (TBI), and later received a matched unrelated donor blood stem cell transplantation. His post-transplant course was complicated by biopsy proven chronic graft-versus-host disease in the gastrointestinal tract requiring daily steroid use. He also developed Cytomegalovirus (CMV) pneumonitis, BK virus-induced hemorrhagic cystitis, Candida glabrata fungemia, and Streptococcus viridans bacteremia-all of which were appropriately treated and resolved.

**Figure 1:**
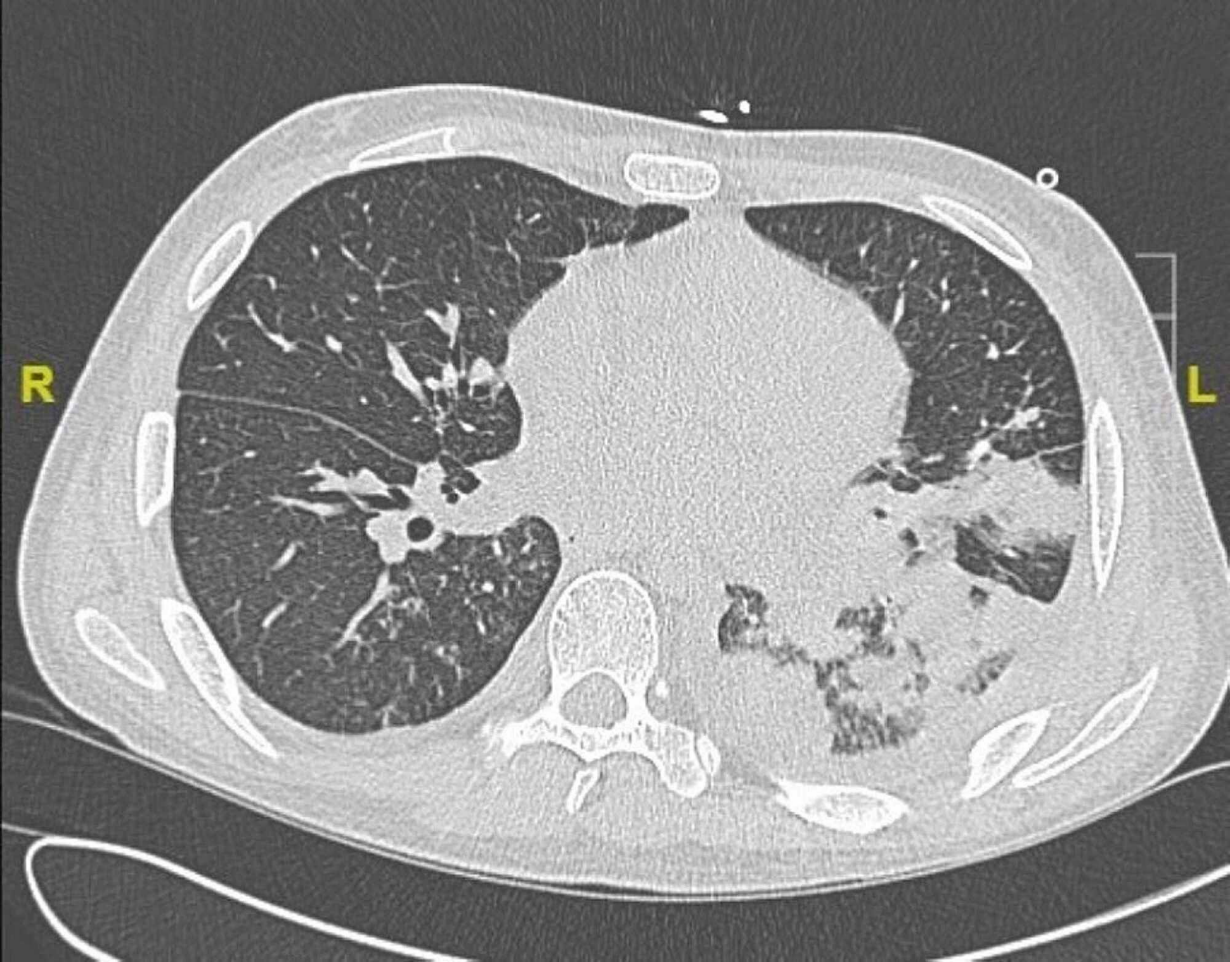
CT chest on admission CT chest obtained on admission to the hospital demonstrating nodular consolidation in the posterior left lower lobe of the lung.

Eleven months after his stem cell transplant, he presented with acute onset of copious blood-tinged phlegm, shortness of breath and left pleuritic chest pain. Upon initial evaluation, the patient was afebrile, respiratory rate was 30 breaths per minute, pulse 180 beats per minute, blood pressure 90/67 mmHg, and oxygen saturation 91% on room air. Broad-spectrum antibiotics including intravenous vancomycin and antifungals were started. The patient later was hypotensive with blood pressure of 70/46 despite aggressive intravenous hydration. Due to septic shock, intravenous norepinephrine drip was started in the intensive care unit. Laboratory studies were significant for pancytopenia with white blood cell count of 2.79 x 10^3^ cells/µL. Intravenous steroids were started given septic shock and patient’s known history of chronic steroid use due to chronic GVHD. Electrolytes and renal function were unremarkable. Arterial blood gas was significant for PaO2 of 64 mmHg. Blood cultures grew methicillin-resistant Staphyloccocus aureus (MRSA). A chest CT scan as seen in Figure 1 showed left lower lobe consolidation in the setting of multiple bilateral cavitary nodules.

The infectious disease service was consulted. Blood cultures grew methicillin resistant Staphyloccocus aureus. Given the patient had not been hospitalized recently, it was determined that he had community-acquired methicillin-resistant Staphylococcus aureus (CA-MRSA) bacteremia. Bronchoscopy was done and was remarkable for the presence of purulent secretions in the tracheobronchial tree. Respiratory culture from bronchoscopy also grew methicillin resistant Staphylococcus aureus. The rest of the infectious workup from the bronchoscopy was unremarkable. A transthoracic echocardiogram was ordered to evaluate for the possibility of endocarditis. The echocardiogram showed a left ventricular ejection fraction of 50%, mild global hypokinesis, and no evidence of vegetation suggestive of endocarditis. After a few days of treatment, the patient had significant clinical improvement and blood cultures were negative. He was discharged with a two week course of intravenous vancomycin which he completed.

**Figure 2:**
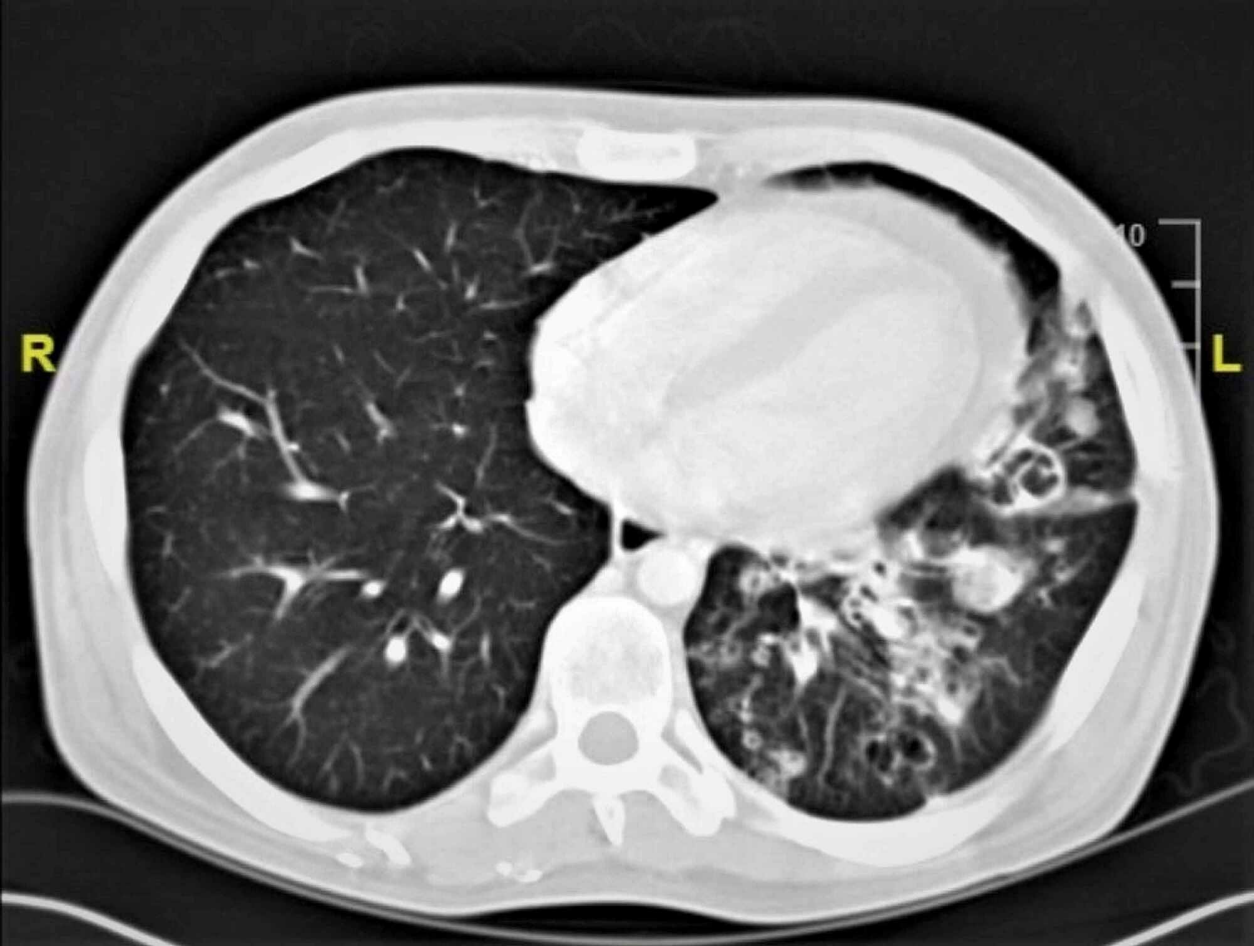
CT chest CT chest shows improvement of the pulmonary consolidation with residual cavitation consistent with a necrotizing process.

Two months later, the patient was admitted to the hospital for fevers, productive cough with green sputum, and shortness of breath. He was found to have recurrent MRSA bacteremia and recurrent MRSA pneumonia. The CT chest findings as seen in Figure 2 showed multiple bilateral cavitary nodules consistent with a necrotizing process.

Initially, intravenous vancomycin was started, however this was later switched to intravenous linezolid. This was done because the patient had recurrent MRSA bacteremia despite prior intravenous vancomycin therapy. Blood cultures were negative after two days of treatment. Transthoracic echocardiogram showed an improved left ventricular ejection fraction of 60-70% and no vegetations were identified. After clinical improvement, the patient was discharged on oral linezolid for six weeks per infectious disease team recommendations. After completing this course he was placed on prophylaxis with oral doxycycline to prevent further CA-MRSA infections.

**Figure 3:**
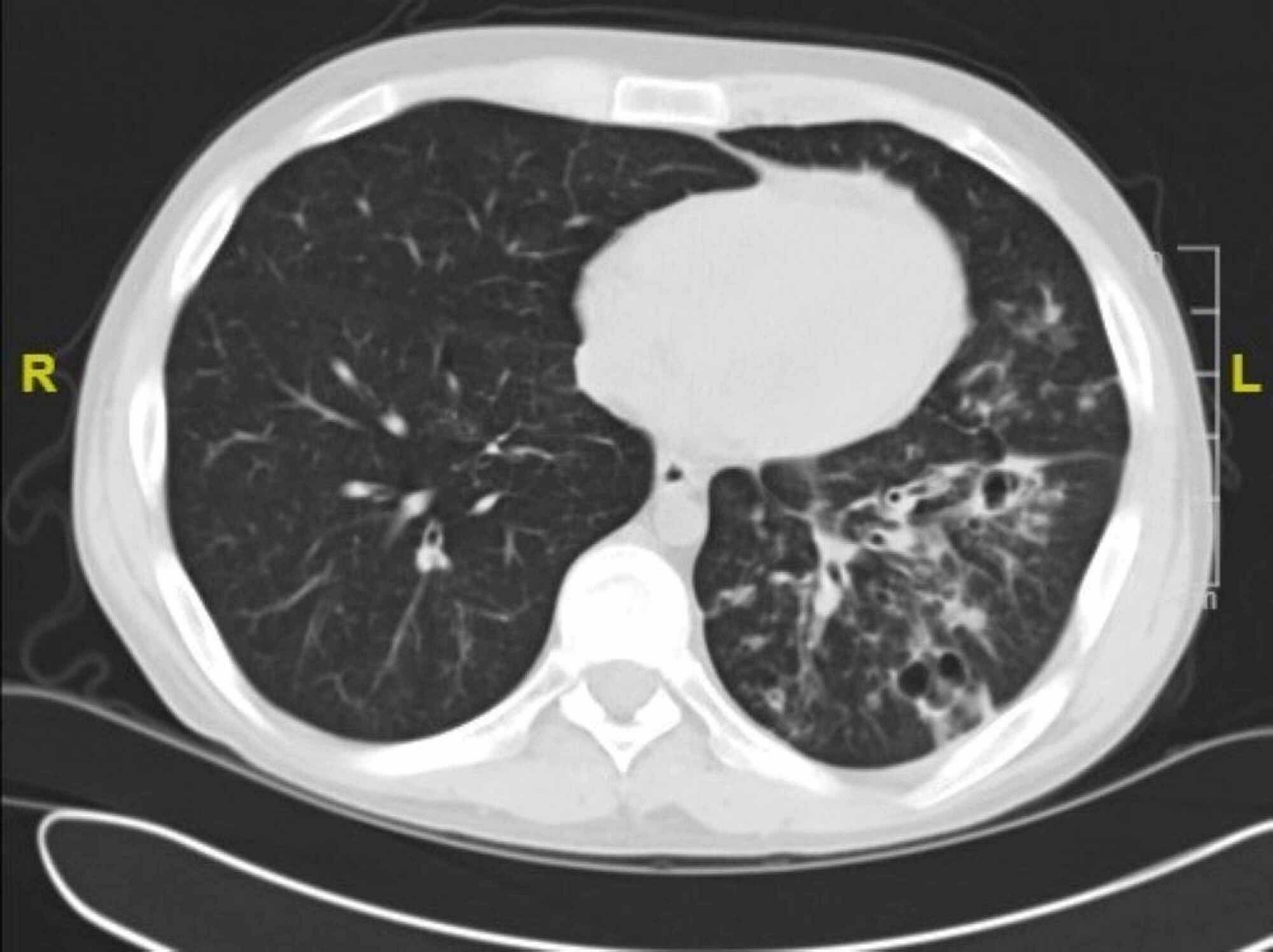
CT chest CT chest demonstrating persistence of cavitary lesions due to prior tissue necrosis.

Six months later, he had a repeat CT of the chest as seen in Figure 3. This CT showed the presence of permanent pneumatoceles from the focal loss of lung parenchyma. 

**Figure 4:**
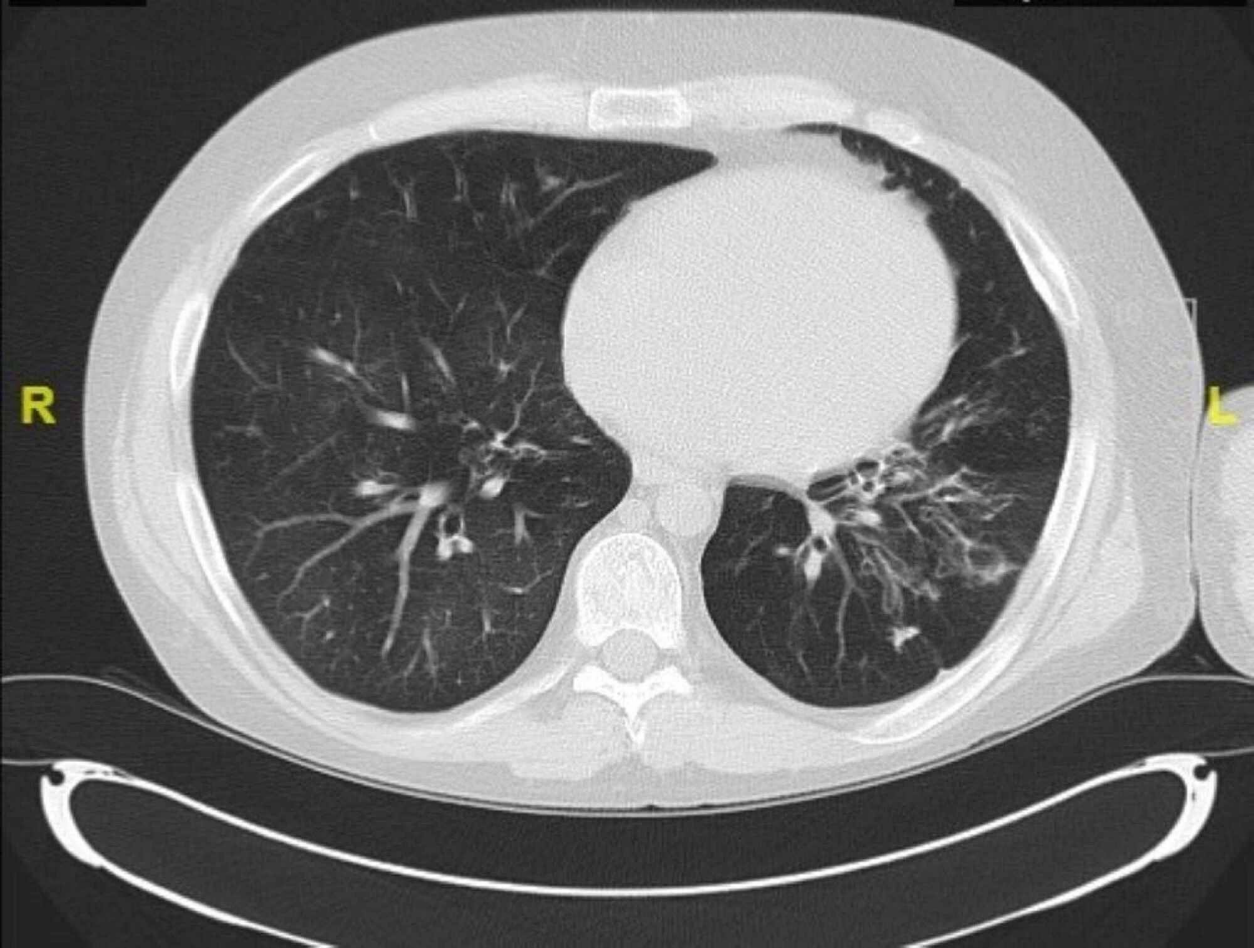
CT chest 2.5 years after initial admission CT chest with findings of permanent cavitary lesions.

Our patient was readmitted on several occasions mainly due to bouts of community-acquired viral pneumonia (adenovirus, parainfluenza, and influenza) along with a secondary Pseudomonas aeruginosa pneumonia. He did not have recurrent CA-MRSA pneumonia. His chest CT scan as seen in Figure 4 from 2.5 years after the original MRSA infection demonstrated bronchiectasis and persistent pneumatoceles from his original infection with CA-MRSA.

His initial recurrent community-acquired pneumonias with secondary MRSA and his recent recurrent episodes of pseudomonas infection suggest the patient was likely chronically colonized.

## Discussion

Necrotizing pneumonia can be caused by multiple pathogens. Our patient initially had necrotizing pneumonia due to CA-MRSA as well as recurrent episodes of pneumonia from both Pseudomonas aeruginosa and CA-MRSA. These organisms can cause necrotizing pneumonia in healthy people, especially in children who have had a recent community-acquired respiratory viral illness. Patients with GVHD have increased susceptibility to bacterial, viral, and fungal infections [2]. Our patient had numerous bouts of viral and bacterial pneumonia, as described above.

The increased susceptibility to bacterial superinfection from viral pneumonia was previously identified, with some authorities theorizing that numerous deaths from the 1918 avian influenza epidemic were due to the secondary bacterial infection that followed the viral illness [3]. Specifically, Streptococcus pneumoniae predominates as the most common bacteria to cause a post-influenza infection, and Staphylococcus aureus is the second most common [4,5]. The damaged airways and local inflammatory response set the stage for the secondary invasion of the aforementioned pathogens. Our patient had prior cytomegalovirus pneumonia that could have made him more susceptible to CA-MRSA pneumonia.

The emergence of CA-MRSA with the marker for virulence Panton-Valentine leukocidin (PVL) has changed the frequency and outcomes of necrotizing pneumonia following a viral respiratory infection. PVL-producing Staphylococcus aureus has been increasing in incidence, particularly in immunocompetent children and young adults, with published mortality rates ranging between 40% and 60% [6,7]. Leukopenia, due to either direct PVL cytotoxicity or by the systemic inflammatory response in the host, has been identified as a direct predictor of lethal outcomes. Our patient was leukopenic upon admission for CA-MRSA pneumonia, likely due to chronic immunosuppression used for chronic GVHD.

In April 2007, the Center for Disease Control published data regarding 10 cases of influenza-associated community-acquired pneumonia caused by CA-MRSA [8]. All 10 patients were immunocompetent and otherwise healthy, and six of them died within a median of 3.5 days after the onset of respiratory symptoms. All the tested strains contained the PVL toxin genes and the staphylococcal cassette chromosome mec type IV resistance gene cassette. In addition, in 2004, the Center for Disease Control reported 11 pediatric deaths due to Staphylococcus aureus following a proven influenza illness [9]. The short duration between onset of illness and death in these studies suggests a synergy between the virus and bacteria, unlike the classic biphasic clinical course of community-acquired pneumonia following influenza. Because CA-MRSA occurs in immunocompetent patients, our patient with a history of donor stem-cell transplantation and chronic GVHD was at a higher risk for morbidity and mortality.

Although there are no standardized treatment guidelines in the setting of chronic GVHD, antibiotic therapy has been shown to reduce mortality, and traditionally vancomycin is used for treating MRSA infections. However, the trend toward administering higher minimum inhibitory concentrations (MIC) of vancomycin has resulted in higher failure rates among patients with infections caused by MRSA who are treated with a MIC greater than 2.0 µg/ml [10,11]. In addition, the resulting necrotic tissue and lack of blood supply impede antibiotic delivery to the infected areas, which allows for the progressive destruction and persistent infection of the pulmonary parenchyma [12]. Several studies have suggested that the combination of a bactericidal agent like vancomycin and a toxin-reducing agent like clindamycin or linezolid may prove beneficial in patients; they have also reported improved survival of MRSA-infected patient when using linezolid compared with vancomycin [13-16]. Given that the necrotizing process is mainly toxin-mediated, the use of antitoxin antibodies has been suggested to be a therapeutic alternative [17].

Staphylococcus aureus produces several toxins, including alpha-hemolysin, beta-toxin, and phenol-soluble modulins, and antibodies against these toxins lead to reduced inflammatory responses [17]. In addition, surgical interventions may be considered for patients whose symptoms fail to improve with antibiotic management [12]. However, there is not enough evidence to support the use of antitoxin antibodies or protein synthesis inhibitors as a standard of care alternative, nor are there clear guidelines regarding optimal indications and timing of surgery. In the setting of known MRSA pneumonia, the American Thoracic Society and the Infectious Diseases Society of America guidelines note a strong evidence base for vancomycin and linezolid over clindamycin [18].

Given that our patient had necrotizing CA-MRSA pneumonia, it is likely that his clinical course improved with the addition of linezolid due to its toxin-reducing properties even though the MRSA was susceptible to vancomycin with a MIC of <1. On initial presentation, our patient was acutely ill with septic shock with MRSA bacteremia and pneumonia. As stated above, leukopenia can lead to poorer outcomes. The use of corticosteroids blunts the massive proinflammatory response that follows invasive CA-MRSA infections. It is likely that in our patient, the use of steroids, which causes leukocytosis through the demargination of neutrophils attached to the endothelial lining of blood vessels, improved his clinical outcome by suppressing infection and his known chronic GVHD. Since our patient had both septic shock and likely adrenal suppression from chronic steroid use due to chronic GVHD, intravenous corticosteroids were indicated. Further studies are needed to extrapolate the use of intravenous corticosteroids in other patients. Lastly, our patient's clinical improvement prevented him from needing surgery, which is often needed in patients with necrotizing pneumonia that is not responsive to medical therapy.

## Conclusions

In this case, we discussed the use of antitoxin and bactericidal antibiotics, as well as the use of corticosteroids as an adjunct therapy to these antibiotics in managing necrotizing pneumonia caused by CA-MRSA in the setting of a patient with chronic GVHD. Given that there are no specific guidelines for treatment in this unique situation, our experience with this patient suggests that the combination of antitoxin antibiotics along with intravenous corticosteroids can be used as an alternative to vancomycin alone. Although this regimen proved beneficial for our patient, further prospective studies are needed to evaluate it as a therapeutic alternative. Studies are needed to better understand the mechanism of this CA-MRSA necrotizing pneumonia and chronic GVHD and to develop guidelines for treatment interventions. 
